# Metabolomic insights into amino acid signatures and pathways associated with osteoporosis in Iranian elderly population

**DOI:** 10.3389/fmed.2025.1515449

**Published:** 2025-05-02

**Authors:** Hojat Dehghanbanadaki, Azin Soltani, Ziba Majidi, Mostafa Rezaei-Tavirani, Gita Shafiee, Afshin Ostovar, Fatemeh Bandarian, Niloufar Najjar, Bagher Larijani, Iraj Nabipour, Patricia Khashayar, Noushin Fahimfar, Farideh Razi

**Affiliations:** ^1^Metabolomics and Genomics Research Center, Endocrinology and Metabolism Molecular-Cellular Sciences Institute, Tehran University of Medical Sciences, Tehran, Iran; ^2^School of Medicine, Tehran University of Medical Sciences (TUMS), Tehran, Iran; ^3^Metabolic Disorders Research Center, Endocrinology and Metabolism Molecular-Cellular Sciences Institute, Tehran University of Medical Sciences, Tehran, Iran; ^4^Department of Medical Laboratory Science, School of Allied Medical Sciences, Tehran University of Medical Sciences, Tehran, Iran; ^5^Proteomics Research Center, Faculty of Paramedical Sciences, Shahid Beheshti University of Medical Sciences, Tehran, Iran; ^6^Chronic Diseases Research Center, Endocrinology and Metabolism Population Sciences Institute, Tehran University of Medical Sciences, Tehran, Iran; ^7^Osteoporosis Research Center, Endocrinology and Metabolism Clinical Sciences Institute, Tehran University of Medical Sciences, Tehran, Iran; ^8^Endocrinology and Metabolism Research Center, Endocrinology and Metabolism Clinical Sciences Institute, Tehran University of Medical Sciences, Tehran, Iran; ^9^The Persian Gulf Marine Biotechnology Research Center, The Persian Gulf Biomedical Sciences Research Institute, Bushehr University of Medical Sciences, Bushehr, Iran; ^10^International Institute for Biosensing, University of Minnesota, Minneapolis, MN, United States

**Keywords:** amino acid, metabolomic, metabolic pathway, osteoporosis, elderly, BMD

## Abstract

**Background:**

Osteoporosis poses a serious health risk to the elderly, particularly in developing countries like Iran. We aimed to determine the 20-amino acids-signatures and pathways associated with osteoporosis in the Iranian elderly population.

**Methods:**

We analyzed the data from the Bushehr Elderly Health Program (BEHP). In the BEHP cohort, participants aged 50 and older in Bushehr, Iran were selected using a multistage stratified random sampling approach. We used logistic regression, pathway enrichment, and pathway impact analysis to determine the metabolites and pathways altered in osteoporosis. AUC-ROC curve analysis assessed the clinical significance of metabolites in differentiating between osteoporosis and control groups.

**Results:**

This study included 1916 participants (1,097 women and 819 men). In women, glycine, citrulline, serine, and aspartic acid were associated with 27, 25, 23, and 21% higher risk of osteoporosis. In men, tyrosine, leucine, valine, and lysine were related with a 24, 22, 22, and 22% reduction in the risk of osteoporosis, respectively. The most impactful altered metabolite pathway among the osteoporotic individuals was “phenylalanine, tyrosine and tryptophan biosynthesis” in both genders. In women, citrulline had an AUC of 0.63 for distinguishing between individuals with osteoporosis and healthy controls. In men, valine, leucine, and tyrosine showed AUC values of 0.62, 0.61, and 0.61, respectively.

**Conclusion:**

Osteoporosis is associated with altered serum amino acids levels in both men and women. The condition is associated with several altered metabolic pathways, with “phenylalanine, tyrosine, and tryptophan biosynthesis” being the most important one. These metabolite signatures and pathways could be targeted for the prevention and management of osteoporosis in older adults.

## Introduction

Osteoporosis is the most prevalent bone disorder marked by reduced bone density, bone tissue degradation, and disruption to bone architecture. The condition often leads to fragility fractures, which typically occur in the spine and hip (1). The global prevalence of osteoporosis is 19.1% and is more common in developing countries, such as Iran (2). A large-scale study found the age-standardized prevalence of osteoporosis in men over 60 to be 29.6% and in women to be as high as 62.7%. This suggests that osteoporosis poses a serious health risk for the elderly population in Iran (3).

Osteoporosis is often not noticed until a fracture happens. Identifying those at risk of fracture is important as the condition is preventable. Although the gold standard of osteoporosis diagnosis is dual x-ray absorptiometry (DXA) scan, it’s not commonly used (4). Given that many available bone turnover markers have pre-analytical and analytical variability (5), there is a need for more accurate alternatives to improve the early prediction and diagnosis of bone loss.

Metabolomics is a new technology that can analyze metabolic end products in body fluids helping with the diagnosis, while improving our understanding of the pathogenesis of diseases such as osteoporosis (6–9). Amino acids, peptides, and related compounds have been the most commonly identified metabolites linked to low bone mineral density (BMD) (10). There is evidence indicating that certain amino acids linked with the growth and differentiation of osteoblasts (11–13), enhancing collagen formation (14, 15), and serving as signaling molecules in bone cells are advantageous for bone health studies (16, 17). Moreover, it is believed that branched-chain amino acids (BCAAs) are involved in increasing the synthesis of muscle proteins (18, 19). Additionally, any increase in the intake of aromatic amino acids (AAAs) could lead to higher levels of insulin-like growth factor-1 (IGF-1) in circulation, directly affecting the calcium hemostasis (13). This is important because these factors play a critical role in stimulating mature osteoblasts (15) and regulating skeletal growth (20). However, the metabolic process of sulfur-containing amino acids (SAAs) can lead to an endogenous acid load, potentially implicating SAAs in acid-mediated impairment of osteoblast function and stimulating the osteoclast activity, which could result in increased bone resorption and decreased bone mass (21–24).

Some studies have suggested a potential connection between certain circulating amino acid, their metabolites and BMD (25, 26). However, these findings have not been consistent and lack repeatability. Therefore, we have decided to study the correlation between circulating amino acid metabolites and bone health in an Iranian elderly population and determine the metabolic pathways that can explain the observed correlations.

## Materials and methods

### Study population

**Figure 1:**
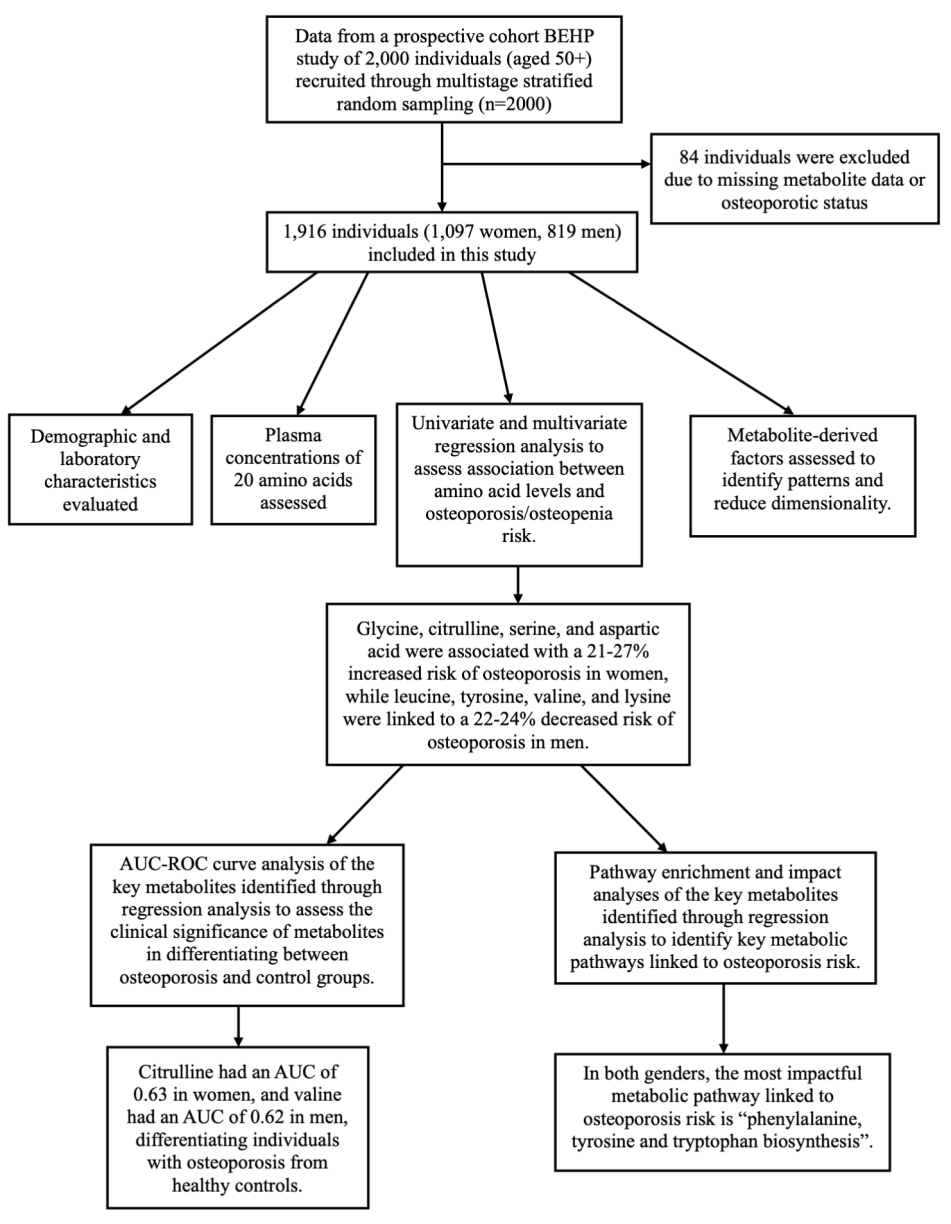
Schematic figure summarizing the study design, analysis context, and key findings.

We used data from the Bushehr Elderly Health Program (BEHP), a community-driven prospective cohort study involving 2,000 individuals aged 50 and above. In BEHP study, the participants were recruited using a multistage stratified random sampling method. Of the 2,000 participants, 84 were excluded due to missing data on metabolite profiles or osteoporotic status, leaving 1,097 women and 819 men in the analysis. The study design and participant selection are shown in Figure 1.

The prevalence of osteoporosis in the BEHP cohort is approximately 28.8%, higher than the national prevalence of 15%. Considering the correlation between predictors and accounting for a 10% attrition rate, a sample size of 1,628 was needed for sufficient statistical power. However, considering the population-based nature of the cohort, a total of 2,000 participants, including 500 with osteoporosis and 1,500 healthy controls, were included in the BEHP cohort to ensure the study had adequate power to detect significant differences (27).

The current study was reviewed and approved by Ethics Committee of the Endocrinology & Metabolism Research Institute (ID code: IR.TUMS.EMRI.REC.1401.088). The details of the protocol for the methodology, data collection, and biochemical measurements of the study had previously been published elsewhere (27, 28).

### DXA and blood tests

As for all participants, bone quantity and quality was evaluated using BMD measurements and trabecular bone score (TBS), respectively. BMD was measured in the lumbar spine, total hip, and femoral neck areas using a dual X-ray absorptiometry (DXA) platform (Hologic Bedford MA USA). Participants were classified based on their T-Score values according to the WHO criteria. Individuals with a T-Score lower than −2.5 were considered osteoporotic and a T-Score between −1 to −2.5 was considered as osteopenia.

After an 8-12-h fasting period, venous blood samples were collected from all individuals and was subjected to hematological, biochemical, and bone turnover markers tests. A portion of the blood and serum was stored at a temperature of −80 degrees Celsius for further analysis in the EMRI BioBank.

### Metabolomics analysis

Amino acids were measured using tandem mass spectrometry with flow injection (FI-MS/MS), a Thermo Scientific Dionex UltiMate 3,000 HPLC system with binary pump (column bypassed) along with a quadrupole mass spectrometer API 320 (SCIEX) equipped with electrospray ionization. Internal standard was added to samples, after which derivatization with acetyl chloride and 1-butanol was performed. The prepared samples were transferred into vials and placed in an automated HPLC sampler. The data were processed using Multiquant software (ABI Sciex). The ratios of metabolite signals to internal standards were used to create calibration curves and calculate analyte concentrations in the materials and QC samples. Detailed analytical performance studies are reported in another study (29).

### Statistical analysis

All statistical analyses were executed in SPSS 19.0. We checked the normality of the continuous variables by Kolmogorov–Smirnov test and reported the normal distributed variables as mean (± standard deviation) and the non-normal distributed variable as median (interquartile range).

The statistical analyses were performed for the women and men study population separately. To test the differences between the three study groups, we used the Chi-square test for categorical variables, the ANOVA and Bonferroni *post hoc* tests for normally distributed continuous variables, and Kruskal-Wallis and Mann–Whitney U tests for non-normally distributed continuous variables.

Benjamini-Hochberg method was applied to adjust the *p*-values from comparisons of metabolites between groups. Metabolite levels were standardized for logistic regression analysis and factor analysis by calculating the Z values of the natural logarithm of their levels. We used logistic regression analysis to determine the odds of developing osteoporosis/osteopenia compared to healthy individuals given each one-unit increase in metabolite concentration. We also conducted a multivariate regression analysis to assess the association between metabolites and osteoporosis/osteopenia risk, adjusting for age, body mass index (BMI), diabetes status, and menopausal status in the women population, and for age, BMI, and diabetes status in the men population.

Subsequently, factor analysis and principal component analysis (PCA) were used to reduce data complexity. Factor analysis grouped correlated amino acids into factors, revealing potential structures linked to osteoporosis, while PCA identified key variables by transforming the data into uncorrelated components. These methods helped simplify the data and highlight the most important factors affecting bone health.

To determine the suitability of data for factor analysis, Kaiser-Meyer-Olkin (KMO) test was used for sampling adequacy whereas Bartlett’s Test of Sphericity was applied for statistical comparison of the correlation matrix with the identity matrix. KMO values of ≥ 0.80 were considered acceptable for factor analysis.

PCA with varimax rotation was performed to condense the metabolites into a more concise set of uncorrelated factors. The factors with eigenvalues above 1.0 and metabolites and loading scores exceeding 0.4 were considered important in the PCA. The score for each factor was computed by adding up the identified standardized metabolites multiplied by their loading matrix, derived from the rotated component matrix using Varimax rotation with Kaiser Normalization. We used univariate and multivariate regression analysis to assess the likelihood of osteoporosis/osteopenia compared relative to controls for each one-unit increase in the factor.

To identify and prioritize the biological pathways most associated with osteoporosis, we have conducted the pathway enrichment and impact analyses based on the altered metabolites between osteoporosis and control groups, as identified through regression analysis. These analyses were performed based on the metabolic Kyoto Encyclopedia of Genes and Genomes (KEGG) database using MetaboAnalyst (Version 5.0). The enrichment ratio was computed based on the observed hits divided by the expected ones.

Finally, to determine the clinical significance of metabolites in distinguishing between osteoporosis and control groups, we performed the receiver operating characteristic (ROC) curve analysis and determine the area under the curve (AUC) of the altered metabolites between osteoporosis and control groups, as identified through regression analysis and evaluate their potential as biomarkers for osteoporosis detection.

## Results

### General characteristics of the study population

The study population was consisted of 1,097 women and 819 men. Among women, 378 (34.46%) individuals had osteoporosis, 488 (44.48%) had osteopenia, and 231 (21.06%) were controls. Among men, 146 (17.83%) had osteoporosis, 431 (52.62%) had osteopenia, and 242 (29.55%) were controls. The osteoporotic people were older than those with osteopenia and healthy individuals. No significant differences were noted in FBS, HbA1c, BMI, and Vitamin D values between the groups (*p*-values ≥ 0.12). Among the female population, only 52 women (5%) were not postmenopausal. The percentage of postmenopausal women was higher in the osteoporotic (98.9%) and osteopenic (95.7%) groups compared to the control group (88.3%, *p*-value < 0.01). General characteristics of the study groups including their BMD, TBS, and bone markers are shown in Table 1. Additionally, Figure 1 presents a schematic overview of the study design, analysis context, and key findings of the study.

**Table 1:** The general characteristics.

Variables	Women (*n* = 1,097)				Men (*n* = 819)			
	Normal (*n* = 231)	Osteopenia (*n* = 488)	Osteoporosis (*n* = 378)	*p*-value	Normal (*n* = 242)	Osteopenia (*n* = 431)	Osteoporosis (*n* = 146)	*p*-value
Age (year)	56.57 ± 5.25	60.32 ± 6.79	66.36 ± 7.54	<0.01	60.34 ± 7.16	62.99 ± 8.53	66.6 ± 9.25	<0.01
FBS (mg/dL)	110.65 ± 36.9	109.28 ± 39.23	108.4 ± 41.42	0.79	115.17 ± 47.33	109.21 ± 38.83	108.03 ± 55.64	0.18
HbA1c (%)	6.53 ± 1.48	6.38 ± 1.43	6.28 ± 1.42	0.13	6.29 ± 1.66	6.16 ± 1.36	6.2 ± 1.71	0.53
BMI (Kg/m^2^)	28.92 ± 4.83	28.2 ± 4.86	28.15 ± 5.02	0.12	28.23 ± 4.9	27.98 ± 4.82	28.23 ± 5.09	0.77
TBS L1-L4	1.38 ± 0.09	1.3 ± 0.09	1.22 ± 0.09	<0.01	1.42 ± 0.09	1.36 ± 0.09	1.28 ± 0.08	<0.01
Lumbar spine BMD (g/cm^2^)	1.07 ± 0.09	0.9 ± 0.08	0.75 ± 0.11	<0.01	1.14 ± 0.12	0.98 ± 0.11	0.79 ± 0.12	<0.01
Lumbar spine T-score	0.2 ± 0.82	−1.31 ± 0.77	−2.74 ± 0.96	<0.01	0.49 ± 1.1	−1.04 ± 0.99	−2.73 ± 1.06	<0.01
Hip BMD (g/cm^2^)	1.03 ± 0.09	0.88 ± 0.08	0.72 ± 0.1	<0.01	1.12 ± 0.1	0.95 ± 0.09	0.81 ± 0.1	<0.01
Hip T-score	0.71 ± 0.7	−0.53 ± 0.69	−1.8 ± 0.86	<0.01	0.58 ± 0.65	−0.57 ± 0.57	−1.47 ± 0.68	<0.01
Femoral neck BMD (g/cm^2^)	0.85 ± 0.08	0.7 ± 0.07	0.55 ± 0.08	<0.01	0.91 ± 0.09	0.73 ± 0.08	0.61 ± 0.09	<0.01
Femoral neck T-score	0 ± 0.73	−1.38 ± 0.64	−2.67 ± 0.73	<0.01	−0.15 ± 0.65	−1.43 ± 0.56	−2.32 ± 0.69	<0.01
BAP (μg/L)	11.91 ± 4.3	13.54 ± 5.06	14.93 ± 6.17	<0.01	11.71 ± 4.43	13.05 ± 5.31	14.59 ± 5.43	<0.01
TRAP (U/L)	2.24 ± 0.91	2.44 ± 0.89	2.85 ± 1.05	<0.01	2.26 ± 0.74	2.39 ± 0.96	2.81 ± 1.07	<0.01
25 OH Vit D (ng/mL)	29.94 ± 16.22	28.51 ± 15.05	28.69 ± 15.5	0.49	24.09 ± 11.15	24.17 ± 11.55	25.76 ± 15.29	0.34
PTH (pg/mL)	52.44 ± 23.58	53.46 ± 22.22	56.37 ± 30.37	0.12	50.62 ± 21.72	52.64 ± 22.85	56.66 ± 25.75	0.04
Osteocalcin (ng/mL)	20.71 ± 9.02	23.91 ± 12.72	28.9 ± 20.27	<0.01	18.43 ± 5.76	21.75 ± 9.4	27.99 ± 12.62	<0.01
CTx (ng/mL)	0.47 ± 0.25	0.63 ± 2.15	0.63 ± 0.31	0.35	0.41 ± 0.2	0.5 ± 0.25	0.6 ± 0.26	<0.01
P1NP (ng/mL)	57.48 ± 28.39	60.15 ± 29.74	71.15 ± 34.26	<0.01	50.07 ± 23.35	57.2 ± 27.4	71.1 ± 50.12	<0.01
Diabetes, *n* (%)	96 (41.6)	175 (35.9)	117 (31)	0.03	87 (36)	133 (30.9)	37 (25.3)	0.08
Menopause, *n* (%)	204 (88.3)	467 (95.7)	374 (98.9)	<0.01	-	-	-	-

FBS, fasting blood sugar; BMI, body mass index; TBS, trabecular bone score; BMD, bone mineral density; BAP, bone-alkaline phosphatase; TRAP, tartrate-resistant acid phosphatase; PTH, parathyroid hormone; CTx, C-terminal telopeptide of type I procollagen; PINP, N-propeptide of type I procollagen.

### Amino acid profiles in the study population

The comparisons of amino acids in women (Supplementary Table S1) showed lower plasma concentrations of valine in osteoporotic individuals [201.1 μmol/L, IQR = 64.82 compared to those with osteopenia (211.44 μmol/L, IQR = 65.39) and healthy subjects (219.44 μmol/L, IQR = 66.28)] whereas citrulline concentrations was higher in this group [36.99 μmol/L, IQR = 14.05 compared to those with osteopenia (34.84 μmol/L, IQR = 13.47) and healthy individuals (33.32 μmol/L, IQR = 13.17)].

Among men, plasma concentration of leucine was lower in osteoporotics [115.4 μmol/L, IQR = 35.05 compared to those with osteopenia (125.61 μmol/L, IQR = 38.71) and healthy individuals (126.16 μmol/L, IQR = 42.98)]. Additionally, osteoporotic men had lower concentrations of tyrosine (71.28 μmol/L, IQR = 20.15) and valine (207.36 μmol/L, IQR = 75.43) than those with osteopenia (75.69 μmol/L, IQR = 26.82 and 229.73 μmol/L, IQR = 64.97, respectively) and healthy subjects (77.78 μmol/L, IQR = 30.33 and 234.29 μmol/L, IQR = 73.84, respectively). The plasma concentrations of amino acids in each study group are shown in Supplementary Table S1.

### Amino acid levels and osteoporosis risk

Women with higher citrulline levels showed a 55% increased likelihood of developing osteoporosis. This risk was as high as 29% with higher glycine and ornithine levels, and 20% with higher aspartic acid levels. An 18% decrease in osteoporosis risk was observed with increased leucine and tyrosine levels, while a 26% decrease was observed with higher valine levels. Additionally, the likelihood of osteopenia among women were 23% higher with increased citrulline levels and 16% lower with elevated alanine levels. After adjusting for age, BMI, diabetes status, and menopausal status, higher glycine levels were associated with a 27% increased risk of osteoporosis. Additionally, higher levels of citrulline, serine, and aspartic acid were linked to a 25, 23, and 21% elevated risk of osteoporosis in the women population (Table 2).

**Table 2:** Odds of osteopenia and osteoporosis per a one-unit increase in amino acids.

Amino acids (μmol/L)	Study groups	Women				Men			
		Univariate analysis	*p*-value	Multivariate analysis*	*p*-value	Univariate analysis	*p*-value	Multivariate analysis**	*p*-value
Alanine	Osteopenia	**0.84 (0.72, 0.98)**	**0.02**	**0.84 (0.71, 0.98)**	**0.02**	1.03 (0.88, 1.21)	0.71	1.07 (0.91, 1.26)	0.38
	Osteoporosis	0.88 (0.75, 1.03)	0.11	0.86 (0.71, 1.04)	0.11	0.88 (0.72, 1.08)	0.23	0.95 (0.77, 1.18)	0.65
Aspartic Acid	Osteopenia	1.15 (0.98, 1.34)	0.07	1.13 (0.97, 1.32)	0.11	1.04 (0.89, 1.22)	0.62	1.03 (0.88, 1.21)	0.68
	Osteoporosis	**1.20 (1.02, 1.42)**	**0.02**	**1.21 (1.01, 1.46)**	**0.04**	1.11 (0.90, 1.37)	0.31	1.09 (0.88, 1.35)	0.43
Glutamic Acid	Osteopenia	1.08 (0.92, 1.26)	0.33	1.06 (0.90, 1.24)	0.47	0.94 (0.81, 1.11)	0.47	0.96 (0.82, 1.12)	0.59
	Osteoporosis	1.11 (0.95, 1.31)	0.19	1.09 (0.91, 1.32)	0.34	0.86 (0.70, 1.06)	0.15	0.88 (0.71, 1.09)	0.24
Leucine	Osteopenia	0.94 (0.81, 1.10)	0.47	1.02 (0.87, 1.20)	0.77	0.97 (0.82, 1.14)	0.69	1.04 (0.88, 1.22)	0.65
	Osteoporosis	**0.82 (0.69, 0.96)**	**0.01**	0.99 (0.82, 1.20)	0.95	**0.68 (0.55, 0.83)**	**<0.01**	**0.78 (0.63, 0.97)**	**0.02**
Methionine	Osteopenia	1.07 (0.91, 1.25)	0.41	1.07 (0.91, 1.25)	0.40	1.04 (0.89, 1.22)	0.59	1.07 (0.92, 1.26)	0.38
	Osteoporosis	1.07 (0.91, 1.26)	0.42	1.09 (0.90, 1.31)	0.37	0.9 (0.73, 1.10)	0.30	0.96 (0.77, 1.19)	0.69
Phenylalanine	Osteopenia	1.07 (0.92, 1.25)	0.40	1.05 (0.90, 1.23)	0.52	0.96 (0.82, 1.12)	0.60	0.97 (0.82, 1.13)	0.67
	Osteoporosis	1.11 (0.94, 1.31)	0.20	1.08 (0.89, 1.29)	0.44	0.96 (0.78, 1.18)	0.70	0.98 (0.79, 1.21)	0.86
Tyrosine	Osteopenia	0.96 (0.82, 1.12)	0.60	0.98 (0.83, 1.15)	0.82	0.86 (0.73, 1.01)	0.06	0.90 (0.76, 1.05)	0.18
	Osteoporosis	**0.82 (0.70, 0.97)**	**0.02**	0.86 (0.72, 1.04)	0.13	**0.70 (0.56, 0.86)**	**<0.01**	**0.76 (0.61, 0.95)**	**0.01**
Valine	Osteopenia	0.90 (0.77, 1.05)	0.16	0.96 (0.82, 1.14)	0.65	0.99 (0.85, 1.16)	0.91	1.08 (0.91, 1.27)	0.37
	Osteoporosis	**0.74 (0.62, 0.87)**	**<0.01**	0.89 (0.73, 1.08)	0.23	**0.66 (0.53, 0.81)**	**<0.01**	**0.78 (0.62, 0.97)**	**0.02**
Arginine	Osteopenia	1.08 (0.92, 1.26)	0.36	1.09 (0.93, 1.27)	0.30	1.06 (0.90, 1.23)	0.50	1.05 (0.90, 1.24)	0.51
	Osteoporosis	1.13 (0.96, 1.34)	0.13	1.19 (0.99, 1.44)	0.07	1.00 (0.81, 1.22)	0.96	0.98 (0.80, 1.22)	0.88
Citrulline	Osteopenia	**1.23 (1.06, 1.44)**	**<0.01**	1.16 (0.98, 1.37)	0.09	1.07 (0.92, 1.25)	0.38	0.98 (0.83, 1.16)	0.80
	Osteoporosis	**1.55 (1.31, 1.83)**	**<0.01**	**1.25 (1.03, 1.53)**	**0.02**	1.11 (0.90, 1.36)	0.33	0.90 (0.73, 1.12)	0.35
Glycine	Osteopenia	1.07 (0.92, 1.25)	0.37	1.05 (0.89, 1.23)	0.55	**1.23 (1.05, 1.45)**	**0.01**	**1.19 (1.01, 1.4)**	**0.03**
	Osteoporosis	**1.29 (1.09, 1.52)**	**<0.01**	**1.27 (1.05, 1.54)**	**0.01**	**1.23 (1.00, 1.52)**	**0.04**	1.13 (0.91, 1.4)	0.28
Ornithine	Osteopenia	1.09 (0.94, 1.27)	0.25	1.05 (0.90, 1.22)	0.55	1.06 (0.91, 1.24)	0.44	1.05 (0.89, 1.23)	0.58
	Osteoporosis	**1.29 (1.09, 1.52)**	**<0.01**	1.19 (0.98, 1.44)	0.07	1.1 (0.90, 1.36)	0.34	1.06 (0.86, 1.31)	0.57
Proline	Osteopenia	0.96 (0.82, 1.12)	0.57	0.95 (0.81, 1.12)	0.54	1.11 (0.95, 1.30)	0.18	1.11 (0.95, 1.31)	0.18
	Osteoporosis	1.06 (0.90, 1.25)	0.49	1.03 (0.85, 1.24)	0.79	1.02 (0.83, 1.26)	0.84	1.03 (0.83, 1.27)	0.81
Threonine	Osteopenia	0.95 (0.81, 1.11)	0.48	0.99 (0.84, 1.17)	0.93	1.07 (0.91, 1.25)	0.42	1.07 (0.91, 1.25)	0.40
	Osteoporosis	0.92 (0.78, 1.09)	0.33	1.04 (0.86, 1.25)	0.70	0.95 (0.77, 1.17)	0.62	0.95 (0.76, 1.17)	0.60
Serine	Osteopenia	1.06 (0.91, 1.24)	0.43	1.07 (0.91, 1.26)	0.38	1.09 (0.93, 1.28)	0.28	1.08 (0.92, 1.26)	0.34
	Osteoporosis	1.13 (0.96, 1.34)	0.13	**1.23 (1.02, 1.49)**	**0.03**	1.16 (0.94, 1.42)	0.16	1.12 (0.90, 1.39)	0.30
Histidine	Osteopenia	0.99 (0.84, 1.15)	0.87	1.02 (0.87, 1.19)	0.81	0.97 (0.83, 1.14)	0.71	0.99 (0.84, 1.16)	0.87
	Osteoporosis	1.02 (0.87, 1.20)	0.80	1.10 (0.91, 1.32)	0.33	0.83 (0.68, 1.02)	0.07	0.86 (0.70, 1.07)	0.17
Lysine	Osteopenia	0.94 (0.80, 1.10)	0.43	0.96 (0.82, 1.13)	0.63	1.10 (0.94, 1.28)	0.25	1.07 (0.91, 1.25)	0.40
	Osteoporosis	0.92 (0.79, 1.09)	0.34	0.93 (0.77, 1.12)	0.43	0.84 (0.68, 1.04)	0.10	**0.78 (0.63, 0.97)**	**0.02**
Tryptophane	Osteopenia	0.91 (0.77, 1.07)	0.26	0.95 (0.81, 1.12)	0.55	0.92 (0.78, 1.08)	0.29	0.95 (0.80, 1.12)	0.55
	Osteoporosis	0.85 (0.71, 1.01)	0.05	0.94 (0.78, 1.14)	0.55	**0.78 (0.63, 0.95)**	**0.01**	0.85 (0.68, 1.05)	0.13
Asparagine	Osteopenia	1.10 (0.94, 1.29)	0.21	1.10 (0.94, 1.30)	0.23	1.03 (0.88, 1.21)	0.71	1.00 (0.85, 1.17)	0.97
	Osteoporosis	1.15 (0.98, 1.36)	0.09	1.15 (0.95, 1.39)	0.14	0.93 (0.76, 1.14)	0.49	0.87 (0.70, 1.07)	0.18
Glutamine	Osteopenia	0.92 (0.78, 1.07)	0.27	0.93 (0.80, 1.09)	0.38	1.05 (0.90, 1.23)	0.51	1.04 (0.88, 1.22)	0.65
	Osteoporosis	0.92 (0.78, 1.08)	0.28	0.92 (0.76, 1.11)	0.37	0.92 (0.75, 1.13)	0.41	0.87 (0.70, 1.07)	0.19

Reference group: normal. *Adjusted for Age, BMI, Diabetes status, and Menopausal status. **Adjusted for Age, BMI, Diabetes status. The values in bold indicate a statistically significant difference (*p* < 0.05).

In the male population, the risk of developing osteoporosis increased by 23% with higher levels of glycine, while 34% reductions in risk was noted with increased valine, 32% with higher leucine, 30% with elevated tyrosine, and 22% with increased tryptophan levels. Additionally, the likelihood of osteopenia among men were 23% higher with elevated glycine levels. After adjusting for age, BMI, and diabetes status, higher levels of tyrosine, leucine, valine, and lysine were each associated with a 22–24% reduction in the risk of osteoporosis (Table 2).

### Metabolite-derived factors and osteoporosis risk

PCA analysis in the women population resulted in five uncorrelated factors with an eigenvalue higher than one in the scree plot (Supplementary Figure S1a). In the univariate regression model, the odds of developing osteoporosis among women was 7% higher with any increase in factor 1, consisted of aspartic acid, glutamic acid, phenylalanine, and glycine (Odds ratio = 1.07, 95%CI: 1, 1.14, *p*-value = 0.03). 17% higher odds was reported with any increase in factor 4, consisted of arginine, citrulline, ornithine, and tryptophane (Odds ratio = 1.17, 95%CI: 1.07, 1.28, *p*-value<0.01). These factors, however, failed to remain significant after adjusting for age, BMI, diabetes status, and menopausal status (Odds ratio = 1.06, 95%CI: 0.99, 1.15, *p*-value = 0.10 and Odds ratio = 1.11, 95%CI: 1, 1.23, *p*-value = 0.05, respectively, Table 3).

**Table 3:** Factor analyses among women.

Factors		Univariate	*p*-value	Multivariate^*^	*p*-value
Factor 1	Osteopenia	1.04 (0.98, 1.11)	0.18	1.03 (0.97, 1.10)	0.30
	Osteoporosis	**1.07 (1, 1.14)**	**0.03**	1.06 (0.99, 1.15)	0.10
Factor 2	Osteopenia	0.97 (0.93, 1.02)	0.27	0.99 (0.94, 1.03)	0.57
	Osteoporosis	0.95 (0.91, 1.00)	0.05	0.98 (0.93, 1.04)	0.47
Factor 3	Osteopenia	0.99 (0.93, 1.05)	0.75	1 (0.94, 1.06)	0.95
	Osteoporosis	0.99 (0.93, 1.06)	0.86	1 (0.93, 1.08)	0.97
Factor 4	Osteopenia	1.07 (0.98, 1.16)	0.11	1.05 (0.96, 1.14)	0.31
	Osteoporosis	**1.17 (1.07, 1.28)**	**<0.01**	1.11 (1.00, 1.23)	0.05
Factor 5	Osteopenia	1 (0.88, 1.14)	0.98	1.03 (0.90, 1.17)	0.69
	Osteoporosis	1.02 (0.89, 1.16)	0.81	1.11 (0.95, 1.30)	0.18

^*^Adjusted for Age, BMI, Diabetes status, and Menopausal status. Factor 1 consisted of aspartic acid, glutamic acid, phenylalanine, and glycine. Factor 2 consisted of alanine, leucine, methionine, tyrosine, valine, and proline. Factor 3 consisted of histidine, lysine, asparagine, and glutamine. Factor 4 consisted of arginine, citrulline, ornithine, and tryptophane. Factor 5 consisted of threonine and serine.The values in bold indicate a statistically significant difference (*p* < 0.05).

As for the male population, PCA analysis resulted in four uncorrelated factors with an eigenvalue higher than one in the screeplot (Supplementary Figure S1b). The univariate regression model showed odds of developing osteoporosis to be 9% lower with any increase in factor 1, containing alanine, leucine, methionine, tyrosine, valine, and tryptophane (Odds ratio = 0.91, 95%CI: 0.86, 0.96, *p*-value<0.01). This factor, similarly, failed to remain significant after adjusting for age, BMI, and diabetes status (Odds ratio = 0.94, 95%CI: 0.89, 1, *p*-value = 0.05, Table 4).

**Table 4:** Factor analyses among men.

Factors		Univariate	*p*-value	Multivariate^*^	*p*-value
Factor 1	Osteopenia	0.99 (0.95, 1.03)	0.64	1.01 (0.96, 1.05)	0.76
	Osteoporosis	**0.91 (0.86, 0.96)**	**<0.01**	0.94 (0.89, 1.00)	0.05
Factor 2	Osteopenia	1.02 (0.97, 1.07)	0.52	1.01 (0.97, 1.06)	0.57
	Osteoporosis	1.01 (0.95, 1.08)	0.67	1.01 (0.94, 1.08)	0.81
Factor 3	Osteopenia	1.02 (0.96, 1.08)	0.56	1.01 (0.95, 1.07)	0.71
	Osteoporosis	0.95 (0.88, 1.02)	0.16	0.93 (0.86, 1.01)	0.07
Factor 4	Osteopenia	1.06 (0.97, 1.15)	0.22	1.03 (0.94, 1.13)	0.53
	Osteoporosis	1.05 (0.93, 1.18)	0.42	0.99 (0.88, 1.11)	0.83

^*^Adjusted for Age, BMI, Diabetes status. Factor 1 consisted of alanine, leucine, methionine, tyrosine, valine, and tryptophane. Factor 2 consisted of aspartic acid, glutamic acid, phenylalanine, glycine, threonine, and serine. Factor 3 consisted of histidine, lysine, asparagine, and glutamine. Factor 4 consisted of arginine, citrulline, ornithine, and proline.The values in bold indicate a statistically significant difference (*p* < 0.05).

### Enriched metabolic pathways linked to osteoporosis risk

**Figure 2:**
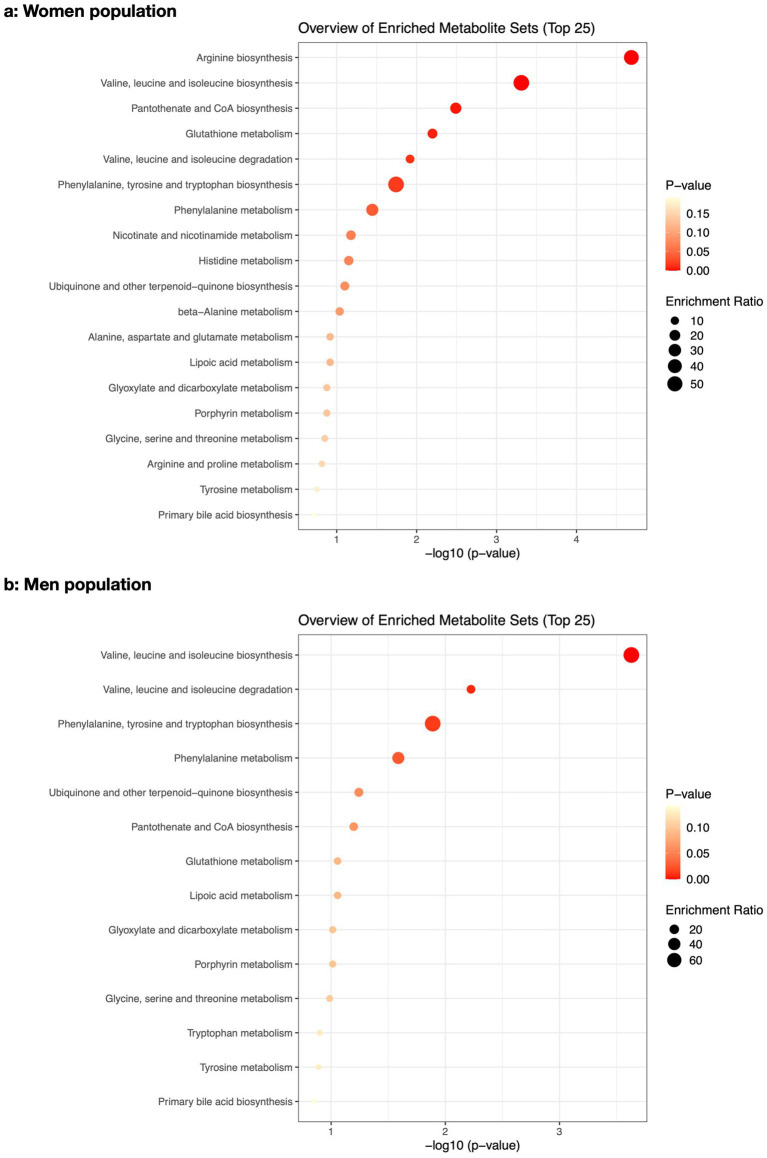
The pathway enrichment analysis of the altered metabolites between osteoporotic and control populations for women **(a)** and men **(b)**.

**Figure 3:**
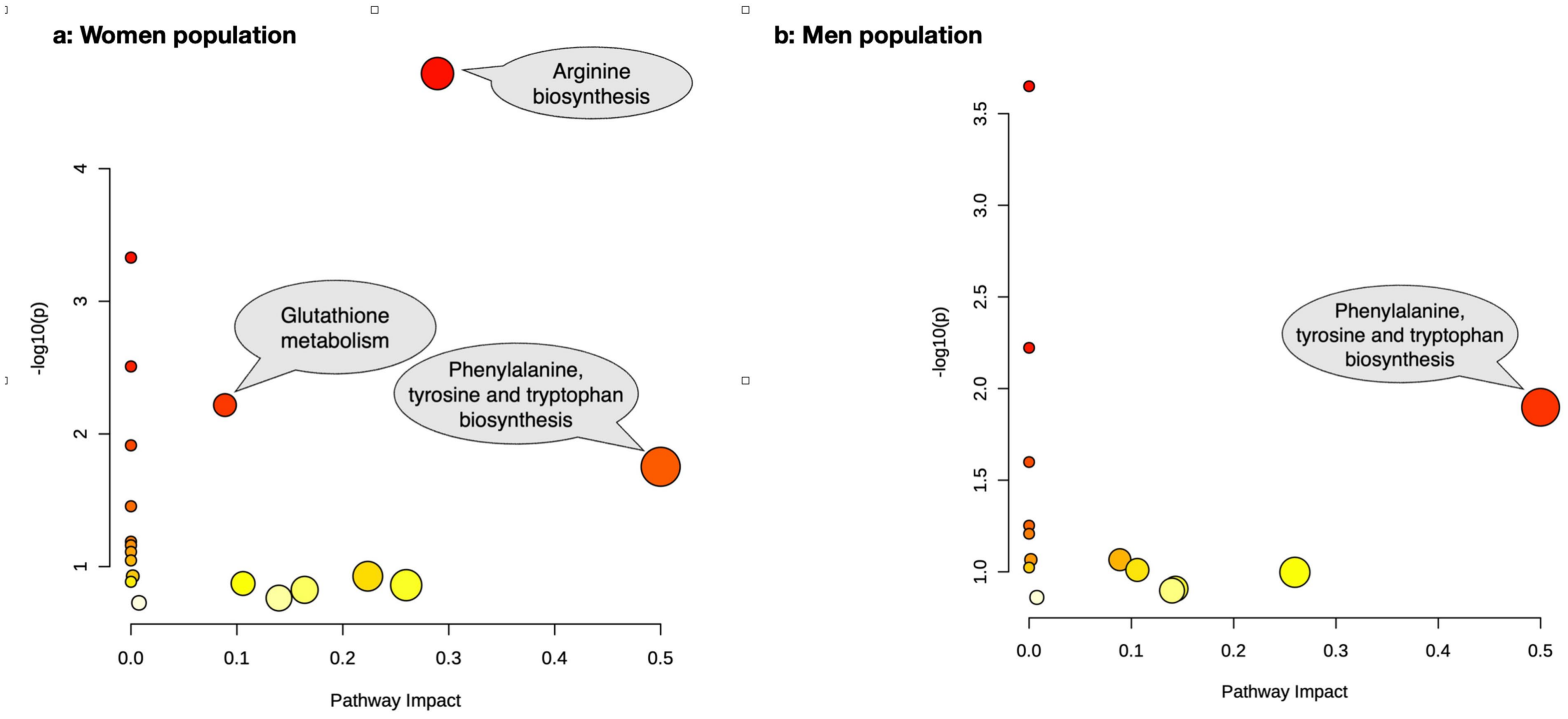
The pathway impact analysis of the altered metabolites between osteoporotic and control populations for women **(a)** and men **(b)**.

The pathway enrichment analysis among women (Figure 2a) demonstrated “arginine biosynthesis,” “valine, leucine and isoleucine biosynthesis,” “pantothenate and CoA biosynthesis,” “glutathione metabolism,” “valine, leucine and isoleucine degradation,” “phenylalanine, tyrosine and tryptophan biosynthesis,” and “phenylalanine metabolism” as the biological processes that best explained altered metabolite differences between osteoporotic and healthy women. Meanwhile, the pathway impact analysis (Figure 3a) demonstrated “phenylalanine, tyrosine and tryptophan biosynthesis,” “arginine biosynthesis,” and “glutathione metabolism” as the most impactful pathways between the same groups, respectively.

In men (Figure 2b), “valine, leucine and isoleucine biosynthesis,” “valine, leucine and isoleucine degradation,” “phenylalanine, tyrosine and tryptophan biosynthesis,” and “phenylalanine metabolism” were the best biological processes explaining altered metabolite differences between osteoporotic and healthy men based on pathway enrichment analysis. The most impactful pathway (Figure 3b) for the same populations was “phenylalanine, tyrosine, and tryptophan biosynthesis.” The details of the pathway enrichment analysis in each gender are illustrated in Supplementary Tables S2, S3.

### ROC analysis of key metabolites in osteoporosis detection

**Figure 4:**
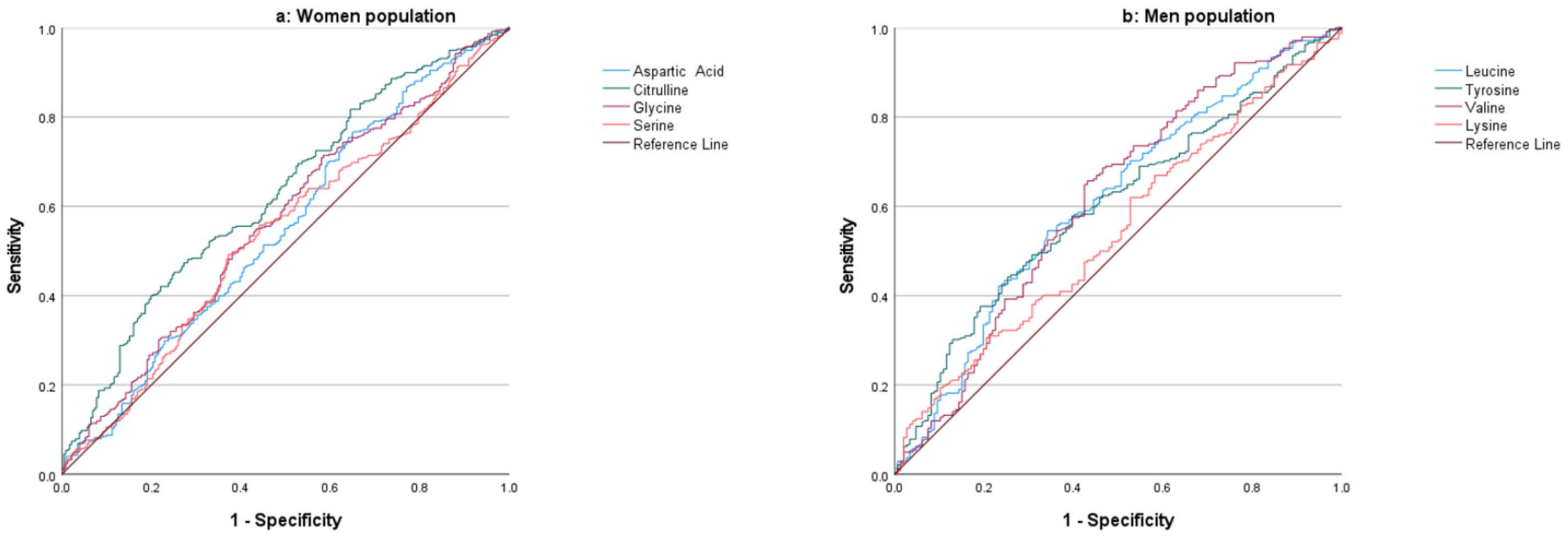
ROC curve analysis of metabolites for distinguishing between osteoporosis and healthy controls in the women **(a)** and men **(b)** populations.

ROC curve analysis (Figure 4a) was performed using metabolites that were statistically different between individuals with osteoporosis and healthy controls based on multivariate analysis. In women, citrulline exhibited the highest AUC of 0.63, demonstrating fair discriminatory power. Glycine showed an AUC of 0.56, while aspartic acid and serine had AUCs of 0.55 and 0.54, respectively, indicating weaker discriminatory abilities.

In men, ROC curve analysis (Figure 4b) revealed that valine had an AUC of 0.62, reflecting fair discrimination between individuals with osteoporosis and healthy controls. Leucine and tyrosine demonstrated similar AUC values of 0.61 and 0.60, respectively, suggesting fair discriminative power. Lysine had the lowest AUC of 0.55, indicating limited ability to distinguish between the two groups.

## Discussion

The present study showed a decrease in valine, leucine, and tyrosine levels in both genders as well as a decrease in tryptophane and lysine in men and alanine in women to be associated with a higher risk of developing osteoporosis/osteopenia. Moreover, increased glycine in both genders along with higher aspartic acid, citrulline, ornithine, and serine in women had a similar effect on the risk of osteoporosis. Any increase in a group of amino acids consisting of aspartic acid, glutamic acid, phenylalanine, and glycine, as well as a group containing arginine, citrulline, ornithine, and tryptophan resulted in a 7 and 17%, increase in the risk of osteoporosis in women, respectively. A surge in a group containing alanine, leucine, methionine, tyrosine, valine, and tryptophan, on the other hand, was found to decrease the risk of osteoporosis. It should be noted that the associations noted in none of these groups remained significant after adjusting for confounding factors. According to our result, the most impactful biological processes that differed between osteoporotic and healthy individuals were phenylalanine, tyrosine, and tryptophan biosynthesis.

Assessing the suitability of data for factor analysis, a KMO value of 0.87 in women and 0.89 in men indicated the adequacy of sample size in both populations. A *p*-value of <0.001 in the Bartlett’s Test of Sphericity for both populations confirmed a significant difference between the correlation and the identity matrices, which similarly indicated the suitability of data for factor analysis.

In line with our study, other research has shown that higher serum levels of valine and leucine are associated with a 17 and 8% lower risk of decreased femoral neck BMD values over a four-year period (25). Moreover, a recent Mendelian randomized study has also found valine as a protective factor for total body BMD (26). In postmenopausal women, higher leucine intake from vegetables was shown to significantly lower the risk of osteoporosis (30). BCAAs, including valine, leucine, and isoleucine, are shown to be crucial for muscle protein synthesis through initiating mRNA translation, which is positively associated with bone strength and density (18, 31).

Based on our findings, the most notable changes in biological processes among individuals with osteoporosis were seen in the biosynthesis of phenylalanine, tyrosine, and tryptophan in both genders, along with arginine biosynthesis and glutathione metabolism in women.

The correlation between AAAs, including tyrosine, tryptophan, and phenylalanine, and BMD values has been investigated in several studies. These studies have shown that BMD and bone histomorphometric variables, such as wall thickness, are positively associated with erythrocyte tryptophan content in men (32). Tryptophan was correlated with lower risk of osteoporosis and the likelihood of a ten-year fracture risk (25). AAAs bind to the calcium-sensing receptors of osteoprogenitor/bone marrow stromal cells, resulting in increased intracellular calcium levels and activated ERK1/2 signaling pathway, both of which leads to osteoblastic proliferation and differentiation (17). AAAs can also suppress osteoclast differentiation and downregulate relevant genes such as vitronectin receptor calcitonin receptor and carbonic anhydrase II (12).

We also confirmed that the arginine biosynthesis was altered in osteoporotic women. Arginine stimulates insulin and IGF-1 secretion, both known as protective factors against osteoporosis, through promoting osteoblast proliferation and collagen synthesis (11, 33). Additionally, the administration of arginine on cultured human osteoblasts is believed to lead to nitric oxide production, which acts as an inhibitor of osteoclastic bone resorption (34).

Glutathione is synthesized from glutamate, cysteine, and glycine. Elevated levels of glutamine have been significantly associated with low BMD values (8). Glutamine may be converted to glutamate, leading to bone resorption through activating glutamate receptors on bone cells, particularly osteoclasts (35). Consistent with our research, elevated serum glycine levels are shown to be associated with decreased femoral neck BMD values and increased major osteoporotic bone fractures. Elevated bone resorption results in increased levels of circulating glycine, mainly because 90% of bone matrix proteins consist of collagen, containing glycine as every third amino acid residue. Additionally, hydroxyproline, which is converted to glycine in the kidney, is another common residue found in the collagen triple helix (36–38). While some researchers suggest SAAs such as cysteine to decrease BMD values through acid-mediated impairment of osteoblast function and stimulation of osteoclast activity (21–24), others have identified SAAs as a protective factor (39, 40).

Our analysis revealed significant gender-specific differences in the amino acid profiles between the osteoporosis and control groups, highlighting that the alterations in amino acid profiles due to osteoporosis were distinct for men and women. This finding aligns with previous studies that have reported gender-related variations in metabolite profiles. Specifically, men exhibited significantly higher levels of BCAAs such as valine and leucine, as well as citrulline, phenylalanine, and tyrosine, compared to women. These amino acids are vital for muscle metabolism, protein synthesis, and the regulation of various physiological processes, which may help explain the observed gender differences (41–43). Conversely, women had higher levels of glycine, lysine, and serine—amino acids associated with neurotransmission, collagen synthesis, and cellular metabolism (42). Notably, the differences in amino acid profiles between osteoporosis and control groups were observed separately for men and women, reinforcing the importance of considering gender as a critical factor in metabolic studies. These findings suggest that men and women may experience distinct physiological responses to osteoporosis, potentially influencing the condition’s development and progression. By highlighting these gender-specific insights, we aim to enhance the understanding of metabolic differences, thereby supporting the development of personalized health strategies that address the unique needs of each gender.

The present study has several strengths. First, to the best of our knowledge, this is the first metabolomic study on osteoporosis that is conducted on a large community of the Iranian population. Second, we have evaluated not only the association between single amino acids and BMD values but also the interaction between different amino acids through PCA analysis. Third using pathway enrichment analysis, we have investigated the biological process responsible for altered metabolites in the osteoporotic population. Fourth, the measured levels of metabolites are available and can be easily utilized in future research. Fifth, we have adjusted our results for several confounding factors. The study, however, suffers from certain limitations. Considering the cross-sectional nature of the study, we cannot conclude a causal link between the identified metabolites and osteoporosis. Moreover, the study was conducted solely on the Iranian population, and therefore the generalizability of the results to other populations needs to be tested in future studies. Additionally, we did not evaluate various comorbidities, such as rheumatoid arthritis or pro-inflammatory markers like CRP, which could potentially influence the results. These unmeasured factors represent limitations of our study. However, the findings remain significant, as they provide valuable insights that can guide future research, particularly in exploring the role of amino acids in osteoporosis and the impact of additional variables on these relationships.

## Conclusion

Current study has suggested an association between several amino acids and osteoporosis in elderly Iranian women and men. These amino acids could therefore be targeted for the management of osteoporosis in older adults; they can also be consumed as dietary supplements to help prevent the condition. Their efficacy in either case, however, should be confirmed in future multicentric studies.

## Data Availability

The data analyzed in this study was obtained from Bushehr Elderly Health Program (BEHP), and is restricted to research purposes only, with confidentiality maintained. Requests to access these datasets should be directed to Prof. Farideh Razi at [f-razi@tums.ac.ir].
