# Supplementary material for: Metabolomic insights into amino acid signatures and pathways associated with osteoporosis in Iranian elderly population
Source: Front Med (Lausanne). 2025 May 2;12:1515449. doi: 10.3389/fmed.2025.1515449 (PMC12081454; doi:10.3389/fmed.2025.1515449)
Supplement: Supplementary file 1 [file Table_1.DOCX]

Table Supplementary S1. Plasma concentration of amino acids

| Amino Acids (µmol/L) | Women | | | | Men | | | |
| --- | --- | --- | --- | --- | --- | --- | --- | --- |
|  | Normal | Osteopenia | Osteoporosis | FDR | Normal | Osteopenia | Osteoporosis | FDR |
| Alanine | 441.72 (370.73, 540.04) | 414.2 (360.88, 499.46) | 423.47 (358.04, 518.16) | 0.30 | 424.8 (361.25, 514.66) | 430.27 (354.1, 514.12) | 425.66 (334.67, 490.86) | 0.60 |
| Aspartic Acid | 29.06 (18.84, 41.72) | 32.54 (21.34, 43.92) | 31.46 (22.43, 44.59) | 0.20 | 31.44 (21.8, 42.04) | 30.74 (21.22, 41.7) | 31.83 (23.29, 43.3) | 0.67 |
| Glutamic Acid | 94.34 (73.09, 118.53) | 97.52 (78.58, 120.72) | 97.2 (77.58, 124.53) | 0.59 | 103.55 (86.55, 129.81) | 102.85 (81.54, 127.1) | 102.4 (83.82, 124.26) | 0.62 |
| Leucine | 109.77 (93.77, 130.23) | 109.75 (93.6, 128.71) | 104.85 (90.26, 125.59) | 0.12 | 126.16 (106.43, 149.41) | 125.61 (105.47, 144.18) | 115.4 (96.41, 131.46) | <0.01 |
| Methionine | 19.39 (16.28, 22.51) | 19.44 (16.97, 22.83) | 19.61 (16.8, 22.94) | 0.74 | 22.36 (19.14, 27.14) | 22.22 (19.18, 26.73) | 21.45 (18.68, 25.37) | 0.60 |
| Phenylalanine | 49.37 (39.38, 60.98) | 51.77 (41, 61.58) | 50.66 (41.56, 62.27) | 0.59 | 54.07 (43.03, 64.88) | 52.41 (42.25, 65.55) | 53.94 (44.99, 62.09) | 0.91 |
| Tyrosine | 74.13 (62.64, 86.69) | 73.59 (61.97, 86.53) | 70.57 (61, 82.74) | 0.12 | 77.78 (65.23, 95.56) | 75.69 (63.82, 90.64) | 71.28 (62.54, 82.69) | 0.02 |
| Valine | 219.44 (189.22, 255.5) | 211.44 (183.32, 248.71) | 201.1 (174.36, 239.18) | <0.01 | 234.29 (199.05, 272.89) | 229.73 (202.72, 267.69) | 207.36 (177.44, 252.87) | <0.01 |
| Arginine | 61.95 (49.41, 78.58) | 62.94 (53.23, 76.17) | 63.98 (53.12, 77.41) | 0.59 | 62.94 (53.18, 75.79) | 64.7 (53.71, 76.42) | 64.41 (52.45, 74.75) | 0.81 |
| Citrulline | 33.32 (25.57, 38.74) | 34.84 (28.34, 41.81) | 36.99 (30.42, 44.47) | <0.01 | 39.53 (32.04, 46.57) | 40.6 (33, 47.8) | 40.26 (33.04, 51.52) | 0.60 |
| Glycine | 231.57 (183.46, 305.97) | 238.62 (186.88, 308) | 260.96 (200.89, 333.14) | 0.05 | 212.94 (177.41, 261.13) | 226.11 (183.15, 279.68) | 227.8 (196.38, 274.75) | 0.06 |
| Ornithine | 74.98 (60.23, 93.37) | 75.56 (63.78, 91.38) | 79.09 (64.99, 95.81) | 0.09 | 79.95 (65.18, 94.79) | 81.12 (66.15, 97.02) | 81.45 (69.24, 96.12) | 0.60 |
| Proline | 229.71 (181.64, 287.89) | 228.34 (179.04, 288.82) | 232.48 (191.8, 299.13) | 0.55 | 254.64 (209.39, 319.92) | 265.05 (214.09, 332.46) | 267.48 (208.45, 342.65) | 0.60 |
| Threonine | 189.3 (153.1, 231.4) | 181.4 (148.63, 223.39) | 181.6 (152.3, 218.48) | 0.59 | 196.4 (151.83, 234.14) | 196.2 (165.2, 243.3) | 192.03 (156.53, 239.85) | 0.60 |
| Serine | 113.22 (93.64, 145.97) | 117.94 (91.22, 151.42) | 122.29 (94.92, 150.2) | 0.55 | 109.89 (86.59, 138.45) | 116.17 (88.39, 145.39) | 120.17 (97.74, 140.56) | 0.56 |
| Histidine | 84.83 (69.91, 106.14) | 86.07 (70.08, 104.42) | 84.61 (69.13, 104.55) | 0.95 | 89.86 (75.52, 107.56) | 90.99 (71.55, 109.97) | 86.51 (71.09, 100) | 0.33 |
| Lysine | 111.43 (89.67, 165.02) | 115.53 (89.01, 156.03) | 114.26 (88.02, 154.83) | 0.90 | 116.15 (90.02, 169.29) | 126.97 (96.18, 174.24) | 113.02 (87.77, 144.64) | 0.02 |
| Tryptophane | 38.74 (32.78, 48.24) | 39.26 (31.26, 47.88) | 36.52 (29.66, 45.09) | 0.09 | 41.71 (33.19, 53.08) | 41.95 (31.98, 51.88) | 38.87 (30.39, 47.26) | 0.10 |
| Asparagine | 48.82 (38.66, 61.35) | 51.37 (41.46, 65.23) | 51.51 (40.1, 66.17) | 0.26 | 55.65 (45.5, 72.77) | 57.43 (46.5, 73.22) | 55.11 (44.85, 70.08) | 0.60 |
| Glutamine | 382 (323, 502) | 384 (317, 472.75) | 385 (302, 482.5) | 0.66 | 397 (322.5, 511) | 410 (334, 514) | 385 (327.75, 474) | 0.33 |

All data is in median (IQR). FDR: false discovery rate (adjusted p-value).

Table Supplementary S2**.** Pathway enrichment analysis between osteoporotic women and normal women. The color of each pathway is based on the p-value [-log(p): logarithm of the p-value, Fisher's test p-value].

|  | **Metabolite Set** | **Total** | **Hits** | **Expect** | **P value** | **Holm P** | **FDR** |
| --- | --- | --- | --- | --- | --- | --- | --- |
| ﻿ | Arginine biosynthesis | 14 | 3 | 0.0637 | 2.06E-05 | 0.00165 | 0.00165 |
| ﻿ | Valine, leucine, and isoleucine biosynthesis | 8 | 2 | 0.0364 | 4.90E-04 | 0.0387 | 0.0196 |
| ﻿ | Pantothenate and CoA biosynthesis | 20 | 2 | 0.091 | 0.00324 | 0.253 | 0.0865 |
| ﻿ | Glutathione metabolism | 28 | 2 | 0.127 | 0.00634 | 0.488 | 0.127 |
| ﻿ | Valine, leucine and isoleucine degradation | 39 | 2 | 0.177 | 0.0121 | 0.922 | 0.194 |
| ﻿ | Phenylalanine, tyrosine, and tryptophan biosynthesis | 4 | 1 | 0.0182 | 0.0181 | 1 | 0.241 |
| ﻿ | Phenylalanine metabolism | 8 | 1 | 0.0364 | 0.0359 | 1 | 0.41 |
| ﻿ | Nicotinate and nicotinamide metabolism | 15 | 1 | 0.0682 | 0.0664 | 1 | 0.628 |
| ﻿ | Histidine metabolism | 16 | 1 | 0.0728 | 0.0707 | 1 | 0.628 |
| ﻿ | Ubiquinone and other terpenoid-quinone biosynthesis | 18 | 1 | 0.0819 | 0.0792 | 1 | 0.634 |
| ﻿ | beta-Alanine metabolism | 21 | 1 | 0.0955 | 0.0919 | 1 | 0.668 |
| ﻿ | Alanine, aspartate and glutamate metabolism | 28 | 1 | 0.127 | 0.121 | 1 | 0.705 |
| ﻿ | Lipoic acid metabolism | 28 | 1 | 0.127 | 0.121 | 1 | 0.705 |
| ﻿ | Glyoxylate and dicarboxylate metabolism | 31 | 1 | 0.141 | 0.133 | 1 | 0.705 |
| ﻿ | Porphyrin metabolism | 31 | 1 | 0.141 | 0.133 | 1 | 0.705 |
| ﻿ | Glycine, serine and threonine metabolism | 33 | 1 | 0.15 | 0.141 | 1 | 0.705 |
| ﻿ | Arginine and proline metabolism | 36 | 1 | 0.164 | 0.153 | 1 | 0.72 |
| ﻿ | Tyrosine metabolism | 42 | 1 | 0.191 | 0.176 | 1 | 0.784 |
| ﻿ | Primary bile acid biosynthesis | 46 | 1 | 0.209 | 0.192 | 1 | 0.807 |

Table Supplementary S3**.** Pathway enrichment analysis between osteoporotic men and normal men. The color of each pathway is based on the p-value [-log(p): logarithm of the p-value, Fisher's test p-value].

|  | **Metabolite Set** | **Total** | **Hits** | **Expect** | **P value** | **Holm P** | **FDR** |
| --- | --- | --- | --- | --- | --- | --- | --- |
| ﻿ | Valine, leucine, and isoleucine biosynthesis | 8 | 2 | 0.026 | 2.35E-04 | 0.0188 | 0.0188 |
| ﻿ | Valine, leucine and isoleucine degradation | 39 | 2 | 0.127 | 0.00596 | 0.471 | 0.239 |
| ﻿ | Phenylalanine, tyrosine, and tryptophan biosynthesis | 4 | 1 | 0.013 | 0.0129 | 1 | 0.345 |
| ﻿ | Phenylalanine metabolism | 8 | 1 | 0.026 | 0.0258 | 1 | 0.515 |
| ﻿ | Ubiquinone and other terpenoid-quinone biosynthesis | 18 | 1 | 0.0585 | 0.0572 | 1 | 0.748 |
| ﻿ | Pantothenate and CoA biosynthesis | 20 | 1 | 0.065 | 0.0634 | 1 | 0.748 |
| ﻿ | Glutathione metabolism | 28 | 1 | 0.091 | 0.0878 | 1 | 0.748 |
| ﻿ | Lipoic acid metabolism | 28 | 1 | 0.091 | 0.0878 | 1 | 0.748 |
| ﻿ | Glyoxylate and dicarboxylate metabolism | 31 | 1 | 0.101 | 0.0969 | 1 | 0.748 |
| ﻿ | Porphyrin metabolism | 31 | 1 | 0.101 | 0.0969 | 1 | 0.748 |
| ﻿ | Glycine, serine and threonine metabolism | 33 | 1 | 0.107 | 0.103 | 1 | 0.748 |
| ﻿ | Tryptophan metabolism | 41 | 1 | 0.133 | 0.126 | 1 | 0.796 |
| ﻿ | Tyrosine metabolism | 42 | 1 | 0.136 | 0.129 | 1 | 0.796 |
| ﻿ | Primary bile acid biosynthesis | 46 | 1 | 0.149 | 0.141 | 1 | 0.805 |

Figure Supplementary S1. The Scree plots of factor analysis for 20 amino acids among women population (a) and among men population (b): eigenvalue distribution and component retention.
